# *δ* ^18^O in the Tropical Conifer *Agathis robusta* Records ENSO-Related Precipitation Variations

**DOI:** 10.1371/journal.pone.0102336

**Published:** 2014-07-25

**Authors:** Bjorn M. M. Boysen, Michael N. Evans, Patrick J. Baker

**Affiliations:** 1 Department of Environmental and Primary Resources, State of Victoria, East Melbourne, Victoria, Australia; 2 Department of Geology and Earth System Science Interdisciplinary Center, University of Maryland, College Park, Maryland, United States of America; 3 Department of Forest and Ecosystem Science, Melbourne School of Land and Environment, University of Melbourne, Victoria, Australia; Penn State University, United States of America

## Abstract

Long-lived trees from tropical Australasia are a potential source of information about internal variability of the El Niño-Southern Oscillation (ENSO), because they occur in a region where precipitation variability is closely associated with ENSO activity. We measured tree-ring width and oxygen isotopic composition (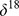
O) of 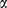-cellulose from *Agathis robusta* (Queensland Kauri) samples collected in the Atherton Tablelands, Queensland, Australia. Standard ring-width chronologies yielded low internal consistency due to the frequent presence of false ring-like anatomical features. However, in a detailed examination of the most recent 15 years of growth (1995–2010), we found significant correlation between 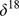
O and local precipitation, the latter associated with ENSO activity. The results are consistent with process-based forward modeling of the oxygen isotopic composition of 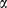-cellulose. The 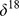
O record also enabled us to confirm the presence of a false growth ring in one of the three samples in the composite record, and to determine that it occurred as a consequence of anomalously low rainfall in the middle of the 2004/5 rainy season. The combination of incremental growth and isotopic measures may be a powerful approach to development of long-term (150+ year) ENSO reconstructions from the terrestrial tropics of Australasia.

## Introduction

The El Niño-Southern Oscillation (ENSO) is one of the leading sources of regional- and global-scale climate variability. A more complete understanding of longer-term, intrinsic variability in ENSO [1] is limited, however, by the absence of direct observations of surface climate before the second half of the 20th century; this is particularly true in the southwest Pacific [2]–[4] and across the West Pacific Warm Pool. The relationship between ENSO and regional climate variability requires a longer historical context, and paleoclimatic reconstructions provide a means of achieving this goal [3]. However, the most widely used high resolution tropical paleoclimatic archives – corals, speleothems, and tree rings – have significant limitations. Corals and speleothem archives are sparsely distributed and hence the paleodata acquired from them often have limited replication. Trees are a widely distributed terrestrial archive and may provide highly replicated paleodata, but in ENSO-affected tropical regions, may not reliably produce annually resolved tree rings [5], [6].

Tropical and sub-tropical tree species and environments present a challenge for paleoclimatology because they often fail to generate the regular annual patterns of cambial activity and dormancy that produce anatomical features reliably identified as annual growth increments ("tree rings") [7], [8]. Short-duration, transient climatic conditions may lead to opportunistic growth and dormancy cycles, which may masquerade as annual growth increments (so-called "false rings") [9]–[13]. Furthermore, persistently poor growing conditions over long time periods may result in missing annual growth increments ("missing rings") [11], [14]. The presence of false and missing growth increments undermines our ability to date materials accurately and precisely – a necessary precursor for paleoclimatic reconstructions from tropical trees. As a result, annually resolved tree rings may provide long, replicated records in the extratropics, but they are rarely reported in the tropical and sub-tropical regions that are directly influenced by ENSO dynamics [15].

Tropical environments are, however, often defined by pronounced and relatively regular variations in moisture, which can be indirectly observed in the oxygen isotopic composition of the 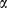-cellulose component of tropical wood [16]. This is because the oxygen isotopic composition of 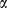-cellulose primarily reflects the isotopic composition of soil moisture, modified by leaf-level evapotranspiration, isotopic exchange between leaf water and unmodified stem water, and biosynthetic fractionation [17], [18]. In the tropics, the isotopic composition of soil moisture is in turn largely determined by the amount of precipitation, because removal of isotopically heavy condensate (at 25°C, the equilibrium fractionation factor for liquid relative to vapor is 1.0092) from a precipitating air mass leaves subsequent precipitation isotopically light [19]–[21]. The "tropical isotope dendroclimatology" hypothesis [16], [22] predicts that sub-annual resolution sampling of tropical trees for isotopic composition can thereby permit detection of an annual cycle in precipitation amount and/or relative humidity, even in trees lacking well-defined annual ring structures [14], [16], [23]–[25]. For tropical trees with growth/dormancy cycles controlled by precipitation seasonality, and which can be therefore also be dated by tree-ring analysis [26]–[29], oxygen isotopic composition of 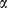-cellulose may reflect interannual variations in precipitation amount and/or relative humidity, complementing information derived from analysis of growth increments, rates and anatomical features [30]–[34].

Northeastern Australia is of particular interest for dendrochronological studies of ENSO variability. Northern Queensland is in close proximity to the Western Pacific Warm Pool, over which ENSO causes changes in the position and strength of large-scale organized convective rainfall [35], [36]. The trees in these areas are thus likely to record ENSO-related interannual variations in local rainfall and relative humidity [22]. If so, then high-quality tree-ring records from this region should have considerable potential as high-fidelity indirect observations of regional ENSO variability. Ogden et al (1981) [37] reported that the tropical forests on the slopes of the Atherton Tablelands may contain more than 100 tree species per hectare, and raised the possibility that these areas may harbor many species with untapped dendrochronological potential. A recent comprehensive survey examining the dendroclimatological potential of ∼180 tropical tree species from northern Queensland identified several candidates, including *Agathis robusta* (Queensland Kauri), a long-lived conifer that reaches enormous sizes (up to 50 m tall and >3 m in diameter at breast height (1.3 m above the ground)), to be of potential value for climate analyses (P.J. Baker, unpublished data; [38]). Despite the well-established crossdating and interpretation of ring-width variations in New Zealand *Agathis australis* [39]–[44], early studies [45], [46] on *A. robusta* in northern Queensland suggested that growth rings were not strictly annual, and that the major factor limiting growth was dry periods. In particular, occasional dry spells led to irregular patterns of cambial dormancy and thereby the formation of false rings. The presence of false rings in this and other tree species from northern Queensland has limited the progress of dendroclimatology in the region (but see [27], [47]).

Here we combine classical dendrochronological techniques with oxygen isotope analyses at seasonal and annual resolution to establish a basis for paleoclimatological studies employing the tropical tree species, *A. robusta*. We assess and discuss the potential of the dual proxy approach for false ring detection and ENSO event reconstruction.

## Materials and Methods

### Study site and species

**Figure 1:**
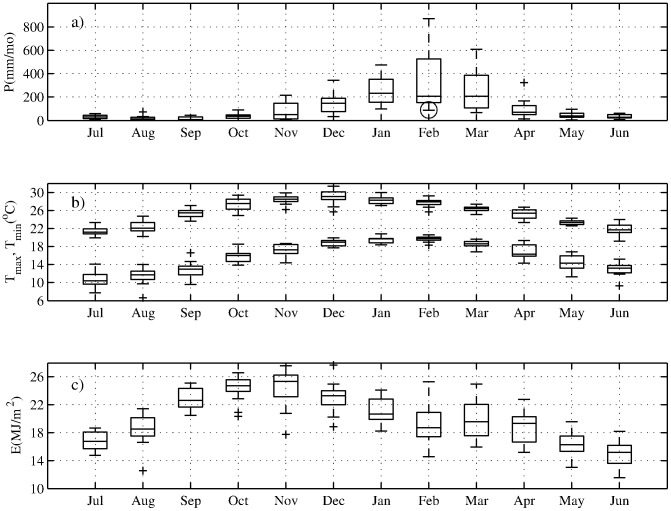
Climatological conditions near the study sites. Monthly averages of precipitation (mm/month; top) daily minimum and maximum temperature (°C; middle), and daily solar exposure (MJ/m^2^; bottom) averaged (as available) from Tinaroo Falls Dam (31075) and Kairi Research Station (31034) for 1996–2010 [61]. Boxes indicate 25th and 75th percentile range of data, with the line within indicating the median; whiskers correspond to approximately the 99% range of data assuming a normal distribution; outliers to this range are plotted as small crosses. The exceptionally dry month of February 2005 is indicated by the large open circle.

Wood sample collections were undertaken, and samples handled, processed and transported in accordance with permits and rules of the Parks and Wildlife Service of the Queensland Department of Environmental Protection, and the Animal and Plant Health Inspection Service of the US Department of Agriculture. No endangered or protected species were sampled in this study. We sampled live *A. robusta* at two locations within Dinden and Danbulla National Parks, situated on the Atherton Tablelands plateau of northern Queensland, Australia. *Agathis robusta* (Araucariaceae) is a large, fast-growing tree with a simple cylindrical growth form and branches that are mainly restricted to the crown. Such characteristics have made this species important for commercial logging, and also make it a prime candidate for dendrochronology. The sites were located at the interface of dry sclerophyll rainforest and wet tropical rainforest. Both National Parks have a mean elevation of ∼700 meters above sea level and are connected by continuous forest. The Atherton Tablelands experience a strong seasonal precipitation cycle (Figure 1), with 85% of annual precipitation occurring between November and April. Warmest temperatures are between October and April, and highest solar exposures are from September through November.

### Dendrochronology

Within each National Park we identified areas in which *A. robusta* was relatively abundant. The sites (in Dinden National Park: 16° 58'45.96"S, 145° 36'11.40"E, and in Danbulla National Park: 17° 8'44.74"S, 145°35'14.49"E) were typically steep, well-drained, south-facing slopes composed of coarse granitic soils, and often contained groups of ∼10–30 *A. robusta* each. We sampled 31 trees at Danbulla and 28 trees at Dinden during early October 2010, before the onset of the wet season. Three cores were taken from each tree at a height of ∼1.2 m using a Haglöf 5.15 mm increment corer. Tree cores were mounted on wooden trays using water-soluble glue for protection and stability during processing. The core surfaces were then sanded using progressively finer grades of sandpaper to obtain a final high quality surface for anatomical analysis, and scanned using an 1800 dpi Microtek 1000XL digital scanner.

Because of the complex nature of anatomical features in the samples, careful visual microscopic examination of the materials was used to check age assignment features. The program COFECHA [48] was used to probabilistically identify dating errors and biases arising from missing and false rings. Following standard practice in dendrochronology, if dating uncertainties for individual series in the sample could not be rectified, those samples were excluded from the site average. Ring-width measurements were made using the image analysis software WinDendro (ver. 2009b, Regent Instruments). The mean interseries correlation (MIC; [49]) of each resulting site composite indicates the level of consistency between all possible pairwise tree core comparisons within a sample set and indicates the level of site-wide (potentially climatic) growth-increment responsiveness.

### Oxygen isotopic analysis

#### Sample preparation

Isotopic sampling was performed at the Department of Geology, University of Maryland, U.S.A. on individual cores from three trees from Dinden National Park. The analysis included dated annual increments corresponding to the period 1995–2010, and leveraged the best dated samples as the basis for isotopic sampling in time. Wood samples for isotopic analysis were obtained from the tree cores using a rotary microtome. Slices of 20 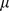
m thickness were taken in succession, and 20 slices were accumulated into one sample for isotopic analysis. This gave 4–8 samples per annual growth increment as determined by dendrochronological analysis described above. For the section of our cores that covered the period 2004–06, we doubled the sampling intensity (every 10th slice) because our dating process had identified the likelihood of a false ring at this time. The more detailed examination of this period enabled us to examine whether the isotopic composition of 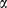-cellulose was consistent with the false-ring interpretation developed *a priori* from the anatomical analysis described previously.

Microtome slices were ground using a small metal rod to increase efficacy of the cellulose extraction chemistry. Cellulose extraction was carried out using the Brendel technique [50] modified for small sample extractions [16], [25]. Samples of 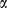-cellulose (mass: 350±20
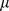
g each) were encapsulated in silver foil and converted to carbon monoxide in a 99.999% helium environment over glassy carbon at 1080°C [51] in a Costech elemental analyzer, passed through a water/CO_2_ trap and a molecular sieve 5A gas chromatographic column to separate the CO analyte from any N_2_ in the sample stream, and then introduced into an Elementar Isoprime mass spectrometer via continuous flow interface. Reported isotopic values reflect within-sample drift correction via monitoring gas measurements, intersample drift correction by periodic measurement of two working standards, and correction for mean and variance bias by within-batch measurement of two 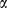-cellulose working standards calibrated to the SMOW reference at known 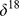
O values of 21.3‰ and 31.0‰, respectively [52], [53]. Long-term precision of measurements, based on replicate analyses of cellulose working standards, are <0.3‰.

#### Isotopic age modeling and compositing

**Figure 2:**
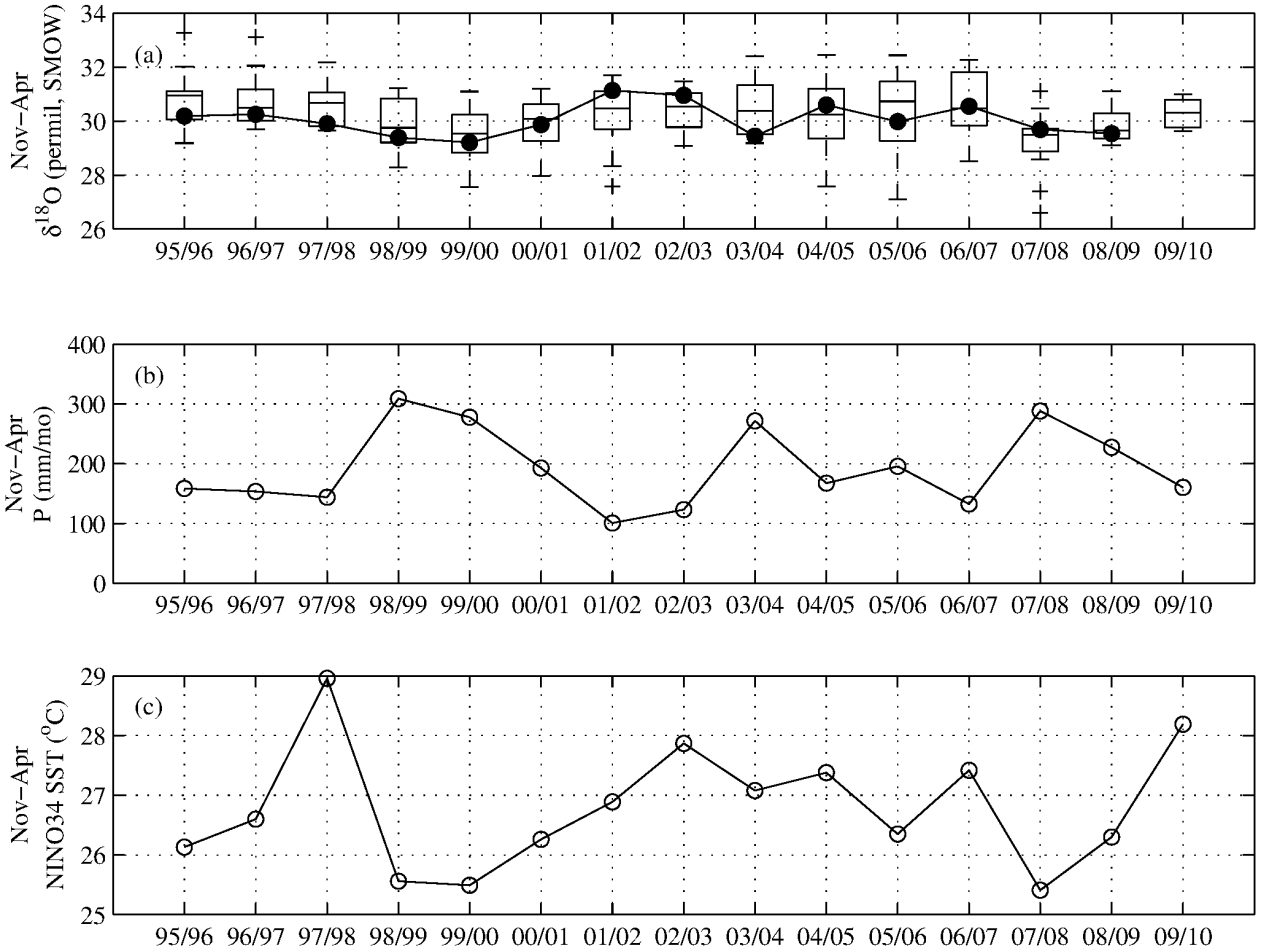
Observed composite 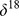
O record from the Atherton Tablelands compared to local precipitation and NINO34 SST index. (a): Box-whisker plots (as described for Fig 1) show the distribution of values for each sample of 12 observations (4 interpolated values per season from each of three trees) in each growing season composite. Calendar age assignments (x-axis) are from the crossdating of the retained dendrochronological subset of tree cores from Dinden National Park (Table 2). Solid circles are process-modeled 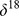
O estimates (see text for details.) (b) November–April average composite precipitation record from Tinaroo Falls and Kairi Research Station (Fig 1). (c) November–April average NINO34 SST.

To develop a composite, annually resolved, calendar-dated oxygen isotope time series, we used the tropical isotope dendroclimatology hypothesis to assign January/February calendar age to isotopic minima within each dendrochronologically dated growth increment, assuming that each minimum in the isotope chronology represents the climatologically average wettest month of the November-April rainy season in northern Queensland (Fig 1; [14], [16], [22], [25]). For each data series, we then linearly interpolated each 4–8 point set of intra-seasonal 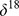
O values to produce a uniform four 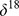
O interpolates within each November-April growing season. This permitted us to composite data across the three sample series on a common intraseasonal time scale. The composite 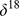
O time series is defined as the median of the interpolated isotopic data within each growing season and across the three replicate data series (Figure 2a).

### Isotopic modeling

To provide a basis for interpretation of the composite 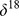
O observations, we simulated the isotopic composition of Dinden 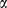-cellulose 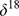
O using the model of Evans (2007) [22]. Modeling inputs are monthly temperature, precipitation amount, and relative humidity, and 18 fixed parameters. Because specific or relative humidity data were not available from the closest Australian Bureau of Meteorology stations (Figure 1), we used the CRU TS3.10.01 [54] estimates of specific humidity and temperature available for 1995–2010 for the 0.5 x 0.5 degree gridpoint closest to the study site, and calculated relative humidity following [55]. The 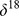
O model is most sensitive to the specification of two parameters that determine the amount effect in precipitation [21], [22]. Accessing all Australian data for oxygen isotopes in precipitation in the GNIP/WISER database [56], we found data for the 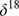
O and amount of precipitation from one tropical station, Darwin (Station 9412000, 12.43°S, 130.87°E, 26 m). The data available from all years (1962–2002 inclusive) were used to construct the statistically significant linear regression (df = 177, r^2^ = 0.22, 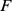 = 50.6, 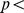
0.001) of precipitation isotopic composition on precipitation amount as: 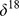
O (‰, SMOW) = −2.56(±0.66)–0.0068(±0.0019)*P (mm/mo) with ±2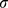 uncertainties on the slope and intercept estimates as indicated. We specified the fraction variance in the simulations associated with precipitation to 0.85 [22], and we set the simulation-mean Nov-April 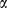-cellulose 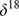
O to that of the composite time series. All other model parameters values were specified as in Tables 1 and 2 of [22], and parameter uncertainty for Monte-Carlo sampling was set to ±40% of parameter values. Because there are many parameters in the model, and the true values of these parameters are largely unknown for this application, we only assess the significance of the linear correlation between the median November–April averaged values from 1000 simulations of the 1995–2009 interval, and the corresponding observed composite medians from the cellulose 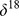
O data series. Interpretation of the correlation of the simulated and observed medians is independent of specified simulated mean and variance, and relies only on the unspecified interannual coherence of the standardized simulation and observational data series. The isotopic modeling represents our best working hypothesis for explaining variance in the composite 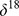
O data series; results are shown in Fig 2a.

**Table 1:** ENSO state for the November-April season between 1995/1996 and 2009/2010.

Warm Phase	Neutral Phase	Cold Phase
1997/1998 (28.96)	2003/2004 (27.08)	2000/2001 (26.26)
2009/2010 (28.19)	2001/2002 (26.89)	1995/1996 (26.13)
2002/2003 (27.87)	1996/1997 (26.60)	1998/1999 (25.56)
2006/2007 (27.42)	2005/2006 (26.35)	1999/2000 (25.49)
2004/2005 (27.38)	2008/2009 (26.30)	2007/2008 (25.41)

Estimated using tercile analysis of the NINO34 SST Index for Nov-Apr 1995/6 through Nov-Apr 2009/2010. Average SST for each seasonal estimate (e.g. Nov 1997 - Apr 1998) is given in parentheses. The NINO34 SST index is an oceanographic indicator of large-scale ENSO activity [58], and is higher (lower) during ENSO warm (cold) phase conditions; warm (cold) phase years are expected to be associated with drier (wetter) conditions in northern Queensland [35], [36].

**Table 2:** Chronology statistics for *A. robusta* at four sites in the Atherton Tablelands, northern Queensland, Australia.

Site	Location	Trees sampled (cores)	Crossdated trees (cores)	MIC
1	Danbulla NP	11 (36)	6 (14)	0.272
2	Danbulla NP	11 (29)	8 (15)	0.298
3	Danbulla NP	9 (28)	5 (12)	0.307
4	Dinden NP	28 (86)	8 (18)	0.276

Mean interseries correlation (MIC) is the average of all possible correlations between width series from individual cores within each site chronology, and is a measure of the quality of the ring width chronology at each site.

### Statistical Analysis

To assess whether isotopic observations, isotopic simulations and meteorological data are consistent with the hypothesized interpretation of the composite 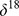
O timeseries, we performed correlation analysis between November–April averages for the common period November 1995–April 2010 (November 1995–April 2009 inclusive for correlation of observed and simulated 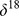
O time series). The null hypothesis is that there is no significant correlation between variables, with significance at the 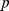 = 0.05 level evaluated as one-tailed for the correlation between observed and modeled 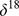
O time series, and two-tailed otherwise.

To assess the response of Dinden precipitation amount and composite 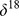
O to ENSO state, we also performed Type I analyses of variance (ANOVA) on November-April averages of monthly precipitation averaged from two nearby Australian Bureau of Meteorology observing stations (Figs 1a, 2b), and on observed composite November-April average cellulose 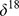
O data (Fig 2a). The treatment groups were defined by the lower, middle and upper terciles of the November-April average NINO34 index, defined as sea surface temperature averaged over the region: 120°W–170°W, 5°N–5°S [57]), for Nov 1995–April 2010 inclusive (Table 1). The NINO34 SST index is an oceanographic indicator of large-scale ENSO activity [58], and is higher (lower) during ENSO warm (cold) phase conditions. The two-tailed null hypotheses for these tests were that there were no observed differences in treatment means among treatment groups, defined as cold, warm and neutral phase ENSO years within the period 1995/6–2009/10 (Table 1).

## Results and Discussion

### Dendrochronology of *A. robusta*

The development of a crossdated tree-ring chronology for *A. robusta* proved to be extremely difficult, because of the widespread presence of putatively false rings. Up to 70% of cores from some sites had to be excluded from the crossdating analysis because it was not possible to accurately assign dates to specific growth increments. A summary of chronology statistics for *A. robusta* from Danbulla National Park (sites 1, 2 & 3) and Dinden National Park (Site 4), is given in Table 2. The difficulties in cross-dating *A. robusta* are indicated by the low mean interseries correlation (MIC) values for each site, despite the exclusion of cores with obvious dating problems. Without additional information, we could not determine whether these features corresponded to within or between growing season growth responses.

### Interpretation of the composite 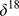
O record

The composite, growing-season averaged 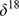
O series is shown together with seasonally averaged station precipitation and NINO34 SST in Figure 2. The 15-year composite 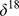
O chronology has an interannual standard deviation of 0.45‰ and within-year composite standard deviation of 0.18‰. Correlations and significances between NINO34 SST (Table 1), Atherton climatological data (Fig 1), observed composite median 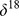
O series (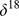
O*_obs_*) and median simulated 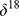
O (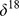
O*_sim_*) are given in Table 3. As expected from the observed influence of ENSO activity on northern Queensland climate, NINO34 SST is significantly correlated with Atherton precipitation (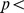
0.01). Consistent with predictions of the tropical isotope dendroclimatology hypothesis, 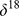
O*_obs_* is negatively correlated with precipitation amount and positively correlated with 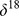
O_*sim*_. The correlation of NINO34 with 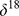
O*_sim_*, however, is not quite statistically significant (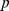 = 0.06). These results are consistent with those of the Type (I) ANOVAs (Table 4), which show that the covariance of 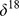
O*_obs_* with ENSO state (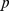 = 0.12) is less significant than the covariance of Atherton precipitation with ENSO state (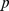 = 0.04). These two results together suggest that additional 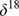
O*_obs_* data, used either to improve growing season composite replication and/or to increase the number of degrees of freedom, should result in increased statistical confidence in our ability to detect ENSO state from the isotopic data.

**Table 3:** Correlation between climatological data, composite median 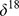
O and simulated median 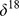
O.

Variable	P	T*_min_*	T*_max_*	S	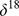 O*_obs_*	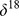 O*_sim_*
NINO34	−0.67**	0.33	−0.47	0.48	0.57*	0.51
P		−0.37	0.11	−0.40	−0.74**	−0.88**
T*_max_*			−0.27	0.69**	0.44	0.53
T*_max_*				−0.32	−0.34	−0.11
S					0.19	0.50
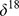 O*_obs_*						0.59*

NINO34 is NINO34 SST; P, T*_max_*, T*_min_*, and S are precipitation, maximum and minimum temperature and solar exposure data compiled from two Atherton meteorological stations (Fig 1). Correlation significances are based on two-tailed t-tests with 13 degrees of freedom (12 degrees of freedom for correlations with 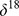
18O*_sim_*). * indicates correlation is significant at 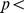
0.05, ** indicates correlation is significant at 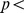
0.01.

**Table 4:** Type (I) ANOVAs: Atherton precipitation and 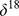
O of *A. robusta*, for the NINO34 sea surface temperature terciles defined in Table 1.

Atherton precipitation					
	SS	df	MS	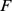	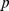
Treatment	25048.04	2	12524.02	4.17	0.04
Error	36052.60	12	3004.38		
Total	61100.64	14			

Test of the null hypothesis that there is no significant difference between the mean precipitation (composite observed 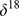
O) observed during above normal, normal, and below normal NINO34 SSTs, 1995/1996–2009/2010 (Table 1). SS  =  sum of squares; MS  =  mean square; df  =  degrees of freedom; 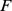  =  F statistic; 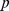  =  p-value.

### Detection of false rings

**Figure 3:**
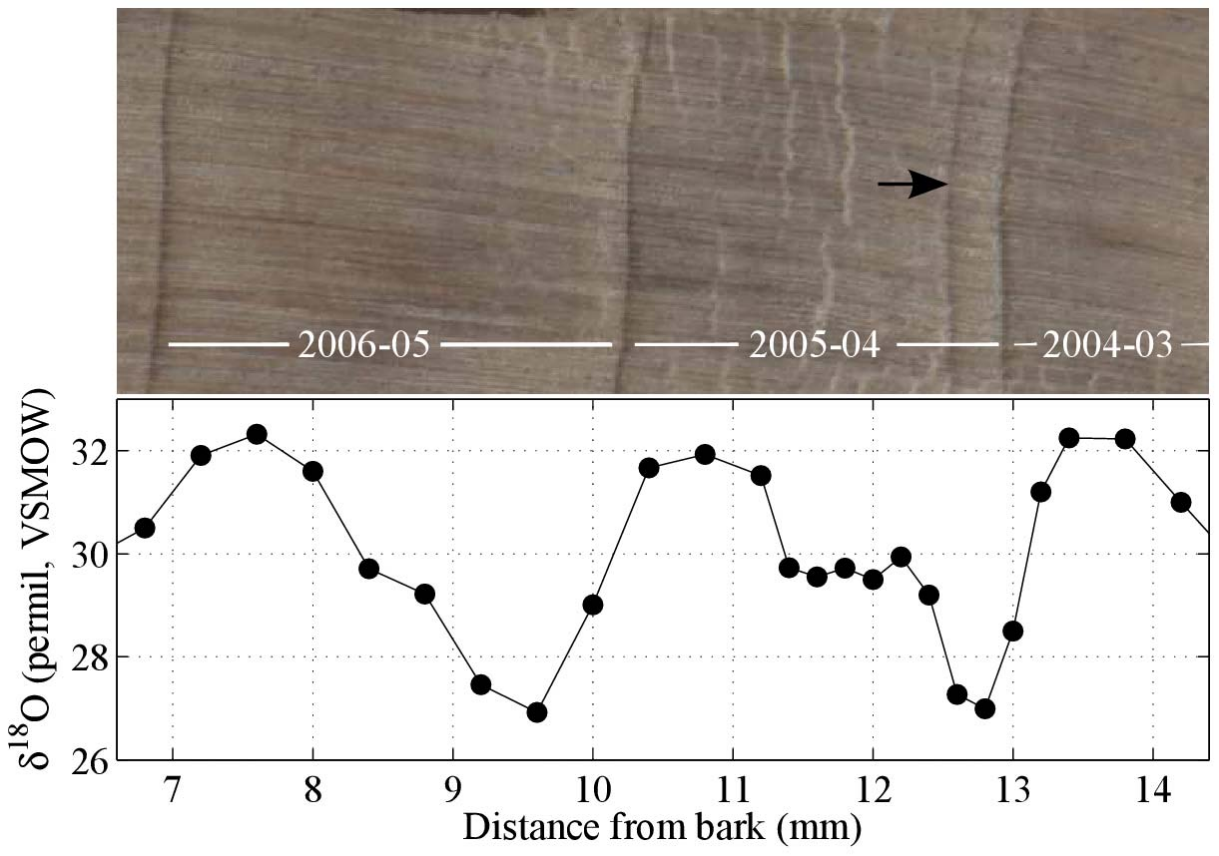
High resolution analysis of a section of *A. robusta*. Top: Section of scan of core A (BB11.17W) from Dinden National Park, including crossdated growth increments (white lines) and suspected false ring (black arrow). Bottom: 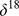
O (‰, SMOW) measured vs. distance from tree bark, scale in millimeters.

Comparison of limited intraseasonal-resolution 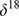
O measurements with wood anatomy suggests the potential for detecting false rings in dendrochronological samples. Figure 3 shows a small section of a tree core spanning two complete growing seasons (2004–05 and 2005–06) for which 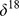
O was measured at a resolution of 8–12 samples per growing season. The 2005–06 growing season shows a simple annual growth ring and an annual cycle of 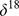
O values consistent with the prediction of the tropical isotope dendroclimatology hypothesis, with minimum 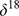
O in the middle of the growth increment, and maximum 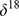
O at the beginning and end of the growing season (Figure 1).

In contrast, the 2004–05 ring presents a problem. In the image of the core (Fig 3, upper panel), there are two anatomically distinct bands of dark, high-density xylem cells in close proximity – one of which, if the isotopic age model hypothesis is correct – is a false ring. In the absence of the isotope data, there is no *a priori* reason to identify either one of these features from 2004–05 as a false ring. The earlier feature might be a false ring if the tree stopped growing near the end of the preceding growing season and then briefly reinitiated growth, due to, for example, an unseasonably late rainfall event. Alternatively, the later feature might be the false ring, if the tree started growing early in the growing season due to an unusually early rainfall event, then stopped due to restored dry season conditions, before the full onset of the wet season, and after which growth was reinitiated. The timing and amount of precipitation delivered during the 2003–2004 rainy season was not unusual. However, in February 2005, in the middle of the rainy season, precipitation in the study region was 89.1 mm, much lower than typical (Fig 1, open circle). Dry conditions at this time may have induced sufficient physiological stress in the trees to initiate the cessation of cambial activity and the formation of latewood-like cells. Many studies across a range of climates have shown that, as a consequence of dry conditions, smaller tracheids with thicker cell walls may be formed by the cambium (e.g. [59]). Tracheids that subsequently form when wetter conditions return are larger in diameter and have thinner walls, typical of earlywood [49]. The effect of this sequence of cambial responses to a transient, but relatively intense, climate anomaly is the formation of a false ring.

High resolution isotopic data (Fig 3, lower panel) support the interpretation of this feature as a false ring within the 2004/2005 growth increment. Relative to the 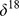
O observations within the 2005/2006 growing season, the 2004/2005 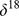
O data appear to indicate an extended period within the growth increment and just following the putative false ring, with isotopic composition intermediate between values indicative of rainy season onset/termination and maximum (Fig 3). This result is consistent with the tropical isotope dendroclimatology hypothesis, which leads us to expect growth through the temporary precipitation hiatus of February 2005 (Fig 1a) within the otherwise normal 2004/2005 rainy season (Fig 2b), albeit with elevated cellulose 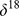
O relative to values observed during rainy season maxima before and after (Fig 3). If the false ring indeed represents February 2005 conditions, the results indicate that about 80% of incremental growth occurred in the last third of the growing season, suggesting that the assumption of linear incremental growth rate within the growing season in this species and environment should be treated with caution [16].

### Prospects

A key limitation in our analyses is the small number of replicates and events studied here. However, the results suggest that the combination of wood anatomical and isotopic analysis might be used to reconstruct tropical paleoclimates from locations and species for which classical methods of dendrochronology alone are unsuccessful. In addition, the superposition of ring width and oxygen isotopic composition measurements may permit the estimation of intraseasonal incremental growth rates in tree species and environments in which growth is episodic. Replication of false ring detection by this dual observational approach would provide additional confidence in our ability to use both increment growth and isotopic composition to more accurately reconstruct wood increment chronology, and thereby subsequently develop improved paleoecological and paleoclimatological interpretations. Conversely, increased confidence in the chronology of tropical species may improve our ability to use the isotopic indicator of rainfall variation to accurately resolve the timing of interannual precipitation variations associated with ENSO activity in the terrestrial tropics. Century-scale oxygen isotope chronologies that crossdate to an acceptable level could be built using long-lived *A. robusta* trees. The results presented here lay the groundwork for that effort.

## Conclusions

If the occurrence of false rings is frequent and undetectable, ring-width chronologies of *A. robusta* from northern Queensland may not be usable as a stand-alone climate indicator. However, the analysis of ring widths and oxygen isotopic composition in this study proved mutually complementary [60]. The small subset of samples that were successfully ring-dated provided the basis for oxygen isotopic analysis of extracted *α*-cellulose, which produced statistically significant correlations with local ENSO-induced variations in precipitation consistent with the tropical isotope dendroclimatology hypothesis. In turn, intraseasonal-resolution oxygen isotope analyses illustrated the basis for more confident detection of false rings in the ring-width data, potentially creating a pathway by which improvements in crossdating accuracy may be made.

We recommend that future studies of *A. robusta* or other species that present ambiguous ring formation use a combination of standard ring-width analysis and oxygen isotopic measurements. By this approach, data developed from these archives may be used to supplement the few existing ENSO reconstructions from the Australasian terrestrial tropics and other regions with climatic variations associated with ENSO activity. We expect that further work should eventually permit development of replicated 150–200 year records.

## References

[1] Collins M, An SI, Cai W, Ganachaud A, Guilyardi E, et al (2010) The impact of global warming on the tropical Pacific Ocean and ENSO. Nat Geo 3: 391–397.

[2] D'Arrigo RD, Cook ER, Wilson RJ, Allan R, Mann ME (2005) On the variability of ENSO over the past six centuries. Geophys Res Lett 32: L03711.

[3] Gergis J, Braganza K, Fowler A, Mooney S, Risbey J (2006) Reconstructing El Niño Southern Oscillation (ENSO) from high-resolution palaeoarchives. J Quat Sci 21: 707–722.

[4] Baker PJ, Palmer JG, D'Arrigo R (2008) The dendrochronology of *Callitris intratropica* in northern Australia: annual ring structure, chronology development and climate correlations. Aust J Botany 56: 311–320.

[5] Dunbar RB, Cole JE, editors (1999) Annual Records of Tropical Systems: Recommendations for Research, 99–1. PAGES/CLIVAR.

[6] Jones PD, Briffa KR, Osborn TJ, van Ommen TD, Vinther BM, et al (2009) High-resolution palaeoclimatology of the last millennium: a review of current status and future prospects. The Holocene 19(1): 3–49.

[7] Worbes M (2002) One hundred years of tree-ring research in the tropics – a brief history and an outlook to future challenges. Dendrchronologia 20: 217–231.

[8] McCarroll D, Loader NJ (2004) Stable isotopes in tree rings. Quat Sci Rev 23: 771–801.

[9] LaMarche VC, Holmes RL, Dunwiddie PW, Drew LG (1979) Chile, volume 2 of *Chronology Series V*. Tucson: University of Arizona.

[10] Stahle DW, D'Arrigo RD, Krusic PJ, Cleaveland MK, Cook ER, et al (1998) Experimental dendroclimatic reconstruction of the Southern Oscillation. AMS Bull 79: 2137–2152.

[11] Pearson SG, Searson MJ (2002) High resolution data from Australian trees. Aust J Botany 50: 431–439.

[12] Anchukaitis KJ, Evans MN, Lange T, Smith DR, Leavitt SW, et al (2008) Consequences of a rapid cellulose extraction technique for oxygen isotope and radiocarbon analyses. Anal Chem 80(6): 2035–2041. 18293945 10.1021/ac7020272

[13] Pearson S, Hua Q, Allen K, Bowman DMJS (2011) Validating putatively cross-dated *Callitris* tree-ring chronologies using bomb-pulse radiocarbon analysis. Aust J Botany 59: 7–17.

[14] Anchukaitis KJ, Evans MN (2010) Tropical cloud forest climate variability and the demise of the Monteverde Golden Toad. Proc Nat Acad Sci 107: 5036–5040. 20194772 10.1073/pnas.0908572107 PMC2841931

[15] Stahle DW, Mushove PT, Cleaveland MK, Roig F, Haynes G (1999) Management implications of annual growth rings in *Pterocarpus angolensis* from Zimbabwe. Forest Ecol Manag 124: 217–229.

[16] Evans MN, Schrag DP (2004) A stable isotope-based approach to tropical dendroclimatology. Geochim et Cosmochim Acta 68(16): 3295–3305.

[17] Roden JS, Lin G, Ehleringer JR (2000) A mechanistic model for interpretation of hydrogen and oxygen ratios in tree-ring cellulose. Geochimica et Cosmochimica Acta 64: 21–35.

[18] Barbour MM, Roden JS, Farquhar GD, Ehleringer JR (2004) Expressing leaf water and cellulose oxygen isotope ratios as enrichment above source water reveals evidence of a Péclet effect. Oecologia 138: 426–435. 14666420 10.1007/s00442-003-1449-3

[19] Dansgaard W (1964) Stable isotopes in precipitation. Tellus 16: 436–468.

[20] Faure G (1986) Isotope Geology. Wiley, 2nd edition.

[21] Gat JR (1996) Oxygen and hydrogen isotopes in the hydrologic cycle. Ann Rev Earth Planet Sci 24: 225–262.

[22] Evans MN (2007) Toward forward modeling for paleoclimatic proxy signal calibration: a case study with oxygen isotopic composition of tropical woods. Geochemistry, Geophysics, Geosystems 8: Q07008.

[23] Verheyden A, Helle G, Schleser GH, Dehairs F, Beeckman H, et al (2004) Annual cyclicity in high-resolution stable carbon and oxygen isotope ratios in the wood of the mangrove tree *Rhizophora mucronata* . Plant, Cell and Environ 27: 1525–1536.

[24] Poussart PF, Schrag DP (2005) Seasonally resolved stable isotope chronologies from northern Thailand deciduous trees. Earth Plan Sci Lett 235: 752–765.

[25] Anchukaitis KJ, Evans MN, Wheelwright NT, Schrag DP (2008) Stable isotope chronology and climate signal calibration in neotropical montane cloud forest trees. J Geophys Res 113: G03030.

[26] Baker PJ, Bunyavejchewin S, Oliver CD, Ashton PS (2005) Disturbance history and historical stand dynamics of a seasonal tropical forest in western Thailand. Ecol Monographs 75: 317–343.

[27] Heinrich I, Weidner K, Helle G, Vos H, Banks JCG (2008) Hydroclimatic variation in Far North Queensland since 1860 inferred from tree rings. Paleog Paleocl Palaeoecol 270: 116–127.

[28] Cook ER, Anchukaitis KJ, Buckley BM, D'Arrigo RD, Jacoby GC (2010) Asian monsoon failure and megadrought during the last millennium. Science 328: 486–489. 20413498 10.1126/science.1185188

[29] Buckley BM, Anchukaitis KJ, Penny D, Fletcher R, Cook ER, et al (2010) Climate as a contributing factor in the demise of Angkor, Cambodia. Proc Nat Acad Sci 107: 6748–6752. 20351244 10.1073/pnas.0910827107 PMC2872380

[30] Vincent L, Pierre G, Michel S, Robert N, Masson-Delmotte V (2007) Tree-rings and the climate of New Caledonia (SW Pacific): Preliminary results from Araucariaceae. Palaeogeogr, Palaeocl, Palaeoecol 253: 477–489.

[31] Ballantyne AP, Baker PA, Chambers JQ, Villalba R, Argollo J (2011) Regional differences in South American Monsoon precipitation inferred from the growth and isotopic composition of tropical trees. Earth Int 15: 1–35.

[32] Brienen RJW, Helle G, Pons TL, Guyot JL, Gloor M (2012) Oxygen isotopes in tree rings are a good proxy for Amazon precipitation and El Niño-Southern Oscillation variability. Proc Nat Acad Sci 109(42): 16957–16962. 23027960 10.1073/pnas.1205977109 PMC3479466

[33] Drew DM, Allen K, Downes GD, Evans R, Battaglia M, et al (2013) Wood properties in a long-lived conifer reveal strong climate signals where ring-width series do not. Tree Phys 33: 37–47. 10.1093/treephys/tps111 23185066

[34] Schollaen K, Heinrich I, Neuwirth B, Krusic PJ, D'Arrigo RD, et al (2013) Multiple tree-ring chronologies (ring width, *δ* ^13^C, and *δ* ^18^O) reveal dry and rainy season signals of rainfall in Indonesia. Quat Sci Rev 73: 170–181.

[35] Ropelewski CF, Halpert MS (1987) Global and regional scale precipitation patterns associated with the El Niño/Southern Oscillation. Mon Wea Rev 114: 2352–2362.

[36] Ropelewski CF, Halpert MS (1989) Precipitation patterns associated with the high index phase of the Southern Oscillation. J Climate 2: 268–284.

[37] Ogden J (1981) Dendrochronological studies and the determination of tree ages in the Australian tropics. J Biogeogr 8: 405–420.

[38] Farjon A (2010) Handbook of the world's conifers. Boston: Brill.

[39] Buckley B, Ogden J, Palmer J, Fowler A, Salinger J (2000) Dendroclimatic interpretation of tree-rings in *Agathis australis* (kauri): 1. Climate correlation functions and master chronology. J Roy Soc New Zealand 30(3): 283–275.

[40] Fowler A, Palmer J, Salinger J, Ogden J (2000) Dendroclimatic interpretation of tree-rings in *Agathis australis* (kauri): 2. Evidence of a significant relationship with ENSO. J Roy Soc New Zealand 30(3): 277–292.

[41] Boswijk G, Fowler A, Lorrey A, Palmer J, Ogden J (2006) Extension of the New Zealand kauri (*Agathis australis*) chronology to 1724 BC. The Holocene 16: 188–199.

[42] Fowler A, Boswijk G, Gergis J, Lorrey AM (2008) ENSO history recorded in *Agathis australis* (kauri) tree rings. Part A: kauri's potential as an ENSO proxy. Int J Clim 28(1): 1–20.

[43] Fowler A (2008) ENSO history recorded in *Agathis australis* (kauri) tree rings. Part B: 423 years of ENSO robustness. Int J Clim 28(1): 21–35.

[44] Fowler AM, Boswijk G, Lorrey AM, Gergis J, Pirie M, et al (2012) Multi-centennial tree-ring record of ENSO-related activity in New Zealand. Nat Clim Change 2(3): 172–176.

[45] Ash J (1983) Tree rings in tropical *Callitris macleayana* F. Muell. Austr J Bot 31: 213–229.

[46] Ash J (1983) Growth rings in *Agathis robusta* and *Araucaria cunninghamii* from tropical Australia. Clim Dyn 31: 269–275.

[47] Heinrich I, Weidner K, Helle G, Vos H, Lindesay J, et al (2009) Wood anatomical features in tree-rings as indicators of environmental change. Clim Dyn 33: 63–73.

[48] Holmes R (1983) Computer assisted quality control in tree-ring dating and measurement. Tree-Ring Bulletin 44: 69–75.

[49] Fritts HC (1976) Tree Rings and Climate. New York: Academic Press.

[50] Brendel O, Iannetta PPM, Stewart D (2000) A rapid and simple method to isolate pure α-cellulose. Phytochemical Analysis 11: 7–10.

[51] Werner RA, Kornexl BE, Roβmann A, Schmidt HL (1996) On-line determination of *δ* ^18^O values of organic substances. Anal Chim Acta 319: 159–164.

[52] Coplen TB, Brand WA, Gehre M, Groning M, Meijer HAJ, et al (2006) New guidelines for *δ* ^13^C measurements. Anal Chem 78: 2439–2441. 16579631 10.1021/ac052027c

[53] Evans MN, Tolwinski-Ward SE, Thompson DM, Anchukaitis KJ (2013) Applications of proxy system modeling in high resolution paleoclimatology. Quat Sci Rev 76: 16–28.

[54] Harris I, Jones PD, Osborn TJ, Lister DH (2014) Updated high-resolution grids of monthly climatic observations – the CRU TS3.10 Dataset. Int J Clim 34: 623–642.

[55] Bolton D (1980) The computation of equivalent potential temperature. Mon Wea Rev 108: 1046–1053.

[56] Birks J (2012) Global Network for Isotopes in Precipitation: The GNIP/WISER Datbase. Technical report, International Atomic Energy Agency. Accessed via Internet: http://www-naweb.iaea.org/napc/ih/index.html.

[57] CPC (2012). Monitoring & Data: Current Monthly Atmospheric and Sea Surface Temperature Index Values. Data accessed via internet: http://www.cpc.ncep.noaa.gov/data/indices/sstoi.indices.

[58] Trenberth KE (1997) The definition of El Niño. Bull Amer Met Soc 78: 2771–2777.

[59] Wimmer R (2006) Wood anatomical features in tree-rings as indicators of environmental change. Dendrchronologia 20: 21–36.

[60] Heinrich I, Allen K (2013) Current issues and recent advances in Australian dendrochronology: Where to next? Geog Res 51: 180–191.

[61] ABOM (2012). Australian Bureau of Meteorology Climate Information. Data accessed via internet: http://www.bom.gov.au/climate/data/.

